# Editorial: Advanced bioinformatic approaches in veterinary virology

**DOI:** 10.3389/fmicb.2024.1354424

**Published:** 2024-02-02

**Authors:** Van Giap Nguyen, Hye Kwon Kim, Woonsung Na

**Affiliations:** ^1^Department of Veterinary Microbiology-Infectious Diseases, Faculty of Veterinary Medicine, Vietnam National University of Agriculture, Hanoi, Vietnam; ^2^Department of Microbiology, Chungbuk National University, Cheongju, Republic of Korea; ^3^Laboratory of Virology, Chonnam National University, Gwangju, Republic of Korea

**Keywords:** bioinformatics, veterinary virology, metagenomics, viral infections, genome

The progress in veterinary virology, driven by pioneering research and high-throughput sequencing, has significantly expanded our understanding of viruses affecting animals, from domestic to wild species. The accumulation of novel virus data through advanced sequencing techniques has revealed previously unknown viruses, providing a wealth of genetic information. However, despite these advancements, there is still a need for in-depth and extensive molecular-based analyses to fully comprehend the complexities of these viral genomes. One of the primary challenges faced by veterinary virologists is extracting meaningful information from the vast amount of collected viral genomic and sequencing data. This includes identifying genetic elements that may be associated with the pathogenicity of these viruses. The quest for understanding the genetic basis of pathogenicity is crucial for developing effective strategies for diagnosis, prevention, and treatment of viral infections in animals.

To address these challenges, experimental studies coupled with advanced bioinformatics methodologies play a pivotal role. These studies extended beyond simple identification and cataloging of viruses; they delve into the molecular intricacies of viral genomes. Bioinformatics tools aid in deciphering the genetic code, identifying potential virulence factors, and understanding the mechanisms by which these viruses interact with host organisms. The integration of experimental studies and advanced bioinformatics in veterinary virology not only enhances our ability to detect and characterize viruses but also opens avenues for discovering new facets of viral pathogenesis. This holistic approach contributes to the development of more targeted and effective interventions in veterinary medicine, ultimately improving the health and wellbeing of animals. As the field continues to advance, the synergy between experimental research and bioinformatics will likely unveil new insights into the diversity, evolution, and pathogenic mechanisms of animal viruses.

Consequently, this Research Topic yielded four original research articles involving 22 authors from two countries including China and the US. The four areas of research cover by this topic include: (i) the applications of viral metagenomics in veterinary virology; (ii) the comparative analysis of viral gene in veterinary virology; (iii) the molecular epidemiology and evolution analysis of veterinary virology and (iv) the recent advancements bioinformatics in the study of emerging of new or re-emerging of previous viruses in livestock.

The first line of this Research Topic indicates recent advancements in bioinformatic approaches, specifically highlighting a hybrid Deep Learning model named DETIRE proposed by Miao et al.. In this study, their work introduces the utilization of graph-based nucleotide sequence embedding for DNA sequence expression enrichment and extracts spatial and sequential features using trained CNN and BiLSTM networks. DETIRE outperforms three of the latest methods (DeepVirFinder, PPR-Meta, and CHEER) in identifying short viral sequences (< 1,000 bp). It was trained on 220,000 sequences of 500 bp subsampled from Virus and Host RefSeq genomes. In summary, DETIRE stands out as a hybrid Deep Learning model that integrates graph-based sequence embedding with CNN and BiLSTM networks, excelling in the identification of short viral sequences in metagenomic data. This research demonstrates the continuous evolution of computational approaches in addressing challenges in veterinary virology and the broader field of genomics.

The Research Topic, delving further into the Metaflye and Canu Assembly Comparison as indicated by Vigil and Aw, provides insights into the assembly of viral contigs using these two bioinformatics tools. Both assemblers identified viral contigs from vertebrates, including *Parvoviridae* and *Poxviridae*. Only Canu successfully assembled viral contigs from dolphin and sea lion fecal samples, matching multiple viral families. It identified viruses associated with both invertebrate and vertebrate hosts, potentially causing mortality events. Consequently, the Metaflye and Canu Assembly Comparison research by Vigil and Aw, contributes to the field of veterinary virology by providing valuable insights into the capabilities of these assemblers in identifying viral contigs from diverse samples. The focus on potential mortality events emphasizes the relevance of such studies for understanding and mitigating the impact of viruses on animal populations.

The second aspect of the research focuses on the application of bioinformatics in veterinary medicine, specifically in the context of Canine Adenovirus 1 (CAdV-1) in mink by Hou J. et al.. The study identifies the presence of Canine Adenovirus 1 (CAdV-1) in mink, suggesting potential cross-species transmission or an existing reservoir in the mink population. The research delves specifically into the bioinformatics analysis of the 100 K protein of CAdV-1, revealing its close relation to strains from Norwegian Arctic fox and Red fox. Bioinformatics analysis further indicates evidence of selective pressure and recombination in the 100 K protein, suggesting evolutionary dynamics and adaptation of the virus over time. This research showcases the application of bioinformatics in characterizing the genetic and protein-level aspects of Canine Adenovirus 1 in mink. The findings contribute to understanding the dynamics of viral evolution, potential cross-species transmission, and the functional role of specific viral proteins. The application of advanced bioinformatics in veterinary medicine allows for a deeper exploration of the intricacies of viral infections in animal populations.

Continuing with the application of informatics, the work by Hou W. et al. represents a significant contribution to the application of bioinformatics in veterinary medicine, specifically in the context of designing a multi-epitope vaccine for Porcine Epidemic Diarrhea Virus (PEDV). The primary objective is to design a multi-epitope vaccine named *rPMEV* for preventing and controlling PEDV infection in pigs. Epitopes are constructed for various immune responses, including cytotoxic T lymphocytes (CTLs), helper T lymphocytes (HTLs), linear B cell epitopes (LBEs), and conformational B cell epitopes (CBEs). Additionally, this multi-epitope design aims to stimulate both cellular and humoral immune responses for comprehensive protection against PEDV. In summary, the work by Hou W. et al. demonstrates the application of informatics in the rational design of a multi-epitope vaccine for PEDV. The use of bioinformatics tools and techniques, along with experimental validations, showcases the potential of computational approaches in advancing vaccine development in veterinary medicine.

Taken together, all the published articles in this Research Topic clearly reflect the interdisciplinary nature of modern veterinary virology, involving deep learning, comparative genomics, epidemiology, and immunoinformatic. The integration of advanced bioinformatics tools not only aids in identifying novel viruses but also contributes to the design of effective vaccines for preventing viral infections in animals. The mentioned studies provide a foundation for further experimental validation and real-world applications in veterinary medicine.

## Author contributions

VN: Writing—original draft, Writing—review & editing. HK: Writing—original draft, Writing—review & editing. WN: Writing—original draft, Writing – review & editing.

